# The Establishment and Diversification of Epidemic-Associated Serogroup W Meningococcus in the African Meningitis Belt, 1994 to 2012

**DOI:** 10.1128/mSphere.00201-16

**Published:** 2016-11-16

**Authors:** Adam C. Retchless, Fang Hu, Abdoul-Salam Ouédraogo, Seydou Diarra, Kristen Knipe, Mili Sheth, Lori A. Rowe, Lassana Sangaré, Absetou Ky Ba, Soumeya Ouangraoua, Dhwani Batra, Ryan T. Novak, Rasmata Ouédraogo Traoré, Xin Wang

**Affiliations:** a Meningitis and Vaccine Preventable Disease Branch, Centers for Disease Control and Prevention, Atlanta, Georgia, USA; b Centre Hospitalier Universitaire Sanou Sourou, Bobo-Dioulasso, Burkina Faso; c Institut National de Recherche en Santé Publique, Bamako, Mali; d Biotechnology Core Facility Branch, Centers for Disease Control and Prevention, Atlanta, Georgia, USA; e Centre Hospitalier Universitaire Yalgado Ouédraogo, Ouagadougou, Burkina Faso; f Laboratoire National de Santé Public, Ouagadougou, Burkina Faso; g Centre Muraz, Bobo-Dioulasso, Burkina Faso; h Centre Hospitalier Universitaire Pédiatrique Charles de Gaulle, Ouagadougou, Burkina Faso; Swiss Federal Institute of Technology Lausanne

**Keywords:** Africa, *Neisseria meningitidis*, disease outbreaks, epidemiology, evolution, meningitis, meningococcus

## Abstract

Epidemics of invasive meningococcal disease (IMD) caused by meningococcal serogroup A have been eliminated from the sub-Saharan African so-called “meningitis belt” by the meningococcal A conjugate vaccine (MACV), and yet, other serogroups continue to cause epidemics. *Neisseria meningitidis* serogroup W remains a major cause of disease in the region, with most isolates belonging to clonal complex 11 (CC11). Here, the genetic variation within and between epidemic-associated strains was assessed by sequencing the genomes of 92 *N. meningitidis* serogroup W isolates collected between 1994 and 2012 from both sporadic and epidemic IMD cases, 85 being from selected meningitis belt countries. The sequenced isolates belonged to either CC175 (*n* = 9) or CC11 (*n* = 83). The CC11 *N. meningitidis* serogroup W isolates belonged to a single lineage comprising four major phylogenetic subclades. Separate CC11 *N. meningitidis* serogroup W subclades were associated with the 2002 and 2012 Burkina Faso epidemics. The subclade associated with the 2012 epidemic included isolates found in Burkina Faso and Mali during 2011 and 2012, which descended from a strain very similar to the Hajj (Islamic pilgrimage to Mecca)-related Saudi Arabian outbreak strain from 2000. The phylogeny of isolates from 2012 reflected their geographic origin within Burkina Faso, with isolates from the Malian border region being closely related to the isolates from Mali. Evidence of ongoing evolution, international transmission, and strain replacement stresses the importance of maintaining *N. meningitidis* surveillance in Africa following the MACV implementation.

**IMPORTANCE** Meningococcal disease (meningitis and bloodstream infections) threatens millions of people across the meningitis belt of sub-Saharan Africa. A vaccine introduced in 2010 protects against Africa’s then-most common cause of meningococcal disease, *N. meningitidis* serogroup A. However, other serogroups continue to cause epidemics in the region—including serogroup W. The rapid identification of strains that have been associated with prior outbreaks can improve the assessment of outbreak risk and enable timely preparation of public health responses, including vaccination. Phylogenetic analysis of newly sequenced serogroup W strains isolated from 1994 to 2012 identified two groups of strains linked to large epidemics in Burkina Faso, one being descended from a strain that caused an outbreak during the Hajj pilgrimage in 2000. We find that applying whole-genome sequencing to meningococcal disease surveillance collections improves the discrimination among strains, even within a single nation-wide epidemic, which can be used to better understand pathogen spread.

## Abstract (precis)

Meningococcal disease (meningitis and bloodstream infections) threatens millions of people across the meningitis belt of sub-Saharan Africa. A vaccine introduced in 2010 protects against Africa’s then-most common cause of meningococcal disease, *N. meningitidis* serogroup A. However, other serogroups continue to cause epidemics in the region—including serogroup W. The rapid identification of strains that have been associated with prior outbreaks can improve the assessment of outbreak risk and enable timely preparation of public health responses, including vaccination. Phylogenetic analysis of newly sequenced serogroup W strains isolated from 1994 to 2012 identified two groups of strains linked to large epidemics in Burkina Faso, one being descended from a strain that caused an outbreak during the Hajj pilgrimage in 2000. We find that applying whole-genome sequencing to meningococcal disease surveillance collections improves the discrimination among strains, even within a single nation-wide epidemic, which can be used to better understand pathogen spread.

## INTRODUCTION

The pathogen *Neisseria meningitidis* is a common cause of meningitis in the 26 sub-Saharan Africa countries of the so-called “meningitis belt,” where small annual epidemics and periodic large epidemics contribute to the highest incidence of meningococcal meningitis in the world (1). *N. meningitidis* serogroup A disease has dramatically decreased since the initiation of mass vaccination with the meningococcal A conjugate vaccine (MACV) in 2010 (2, 3), and yet, affordable conjugate vaccines are not available for other serogroups that have caused epidemics in Africa. *N. meningitidis* serogroup W remains a major cause of meningococcal disease in the region (4).

The first large outbreak of *N. meningitidis* serogroup W disease occurred during the 2000 Hajj to Saudi Arabia (Islamic pilgrimage to Mecca), with over 300 cases reported worldwide (5, 6). *N. meningitidis* serogroup W isolates from this outbreak (hereinafter, the Hajj-related outbreak strain) belonged to clonal complex 11 (CC11), a hyperinvasive lineage typically identified with *N. meningitidis* serogroup C disease (7, 8). All known CC11 *N. meningitidis* serogroup W isolates originate from a single ancestral strain, with some isolates having been collected as early as 1970 (8, 9). CC11 *N. meningitidis* serogroup W isolates have become globally distributed and were reported in meningitis belt countries as early as 1993 (9) but were not reported from epidemics until 2001 (10). Although *N. meningitidis* serogroup W disease caused by both CC11 and CC175 has been reported in meningitis belt countries (11), only *N. meningitidis* serogroup W CC11 has caused epidemics. A large *N. meningitidis* serogroup W epidemic occurred in Burkina Faso during 2002, with 12,587 cases reported (12, 13). Since then, additional *N. meningitidis* serogroup W epidemics have only occurred since 2010 (14, 15), including the CC11 epidemic in Burkina Faso during 2012, with 5,807 cases reported (4, 16, 17).

The occurrence of several CC11 *N. meningitidis* serogroup W epidemics since 2000 raised concerns that the Hajj-related outbreak strain had become established in the meningitis belt after being carried there by returning pilgrims (12, 14, 18). This was of particular concern because the attack rate among returning pilgrims was reported to be as high as 25 per 100,000 population, with a case fatality rate as high as 37% (19), suggesting that the Hajj-related outbreak strain may have been more virulent than other *N. meningitidis* serogroup W strains. Isolates that evolved from this strain continue to be identified around the world, and these strains may have become endemic in regions as distant as South Africa, Turkey, and Europe (8, 20). However, CC11 *N. meningitidis* serogroup W strains that are endemic to the United Kingdom and Chile did not evolve from the Hajj-related outbreak strain (8, 20). While isolates from the 2002 epidemic in Burkina Faso did not evolve from the Hajj-related outbreak strain (12, 20, 21), they are closely related, and it is unclear when these two strains diverged or whether the recent CC11 *N. meningitidis* serogroup W isolates from meningitis belt countries are descended from the Hajj-related outbreak strain.

Here, we applied whole-genome sequencing to analyze the evolution of *N. meningitidis* serogroup W populations in the meningitis belt countries from 1994 to 2012. Isolates collected during the 2012 epidemic in Burkina Faso and the preceding years in the belt were analyzed to identify the origin of the 2012 epidemic *N. meningitidis* serogroup W and the relationship of that epidemic to the 2002 epidemic in Burkina Faso. Isolates collected during the Hajj-related Saudi Arabian outbreak in 2000 and during the following years in Africa were analyzed to evaluate whether the Hajj-related outbreak strain was dispersed to Africa and contributed to later epidemics. We further examined the genetic variation among major lineages to identify loci distinguishing the epidemic-associated isolates from other CC11 *N. meningitidis* serogroup W strains.

## RESULTS

### Overview of phylogenetic diversity.

**FIG 1:**
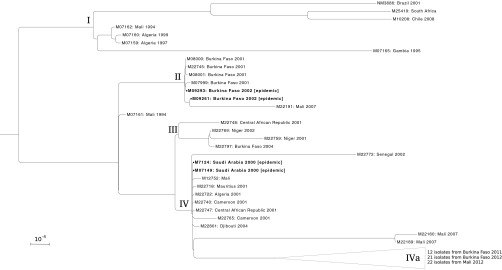
Maximum-likelihood phylogeny of 86 CC11 *N. meningitidis* serogroup W isolates, rooted on NM3683. Branches with less than 85% bootstrap support are collapsed. Scale bar represents one substitution per 10,000 bases in the whole-genome alignment. Isolates are labeled with country and year of isolation if available. Isolates from the 2002 Burkina Faso epidemic and the 2000 Hajj-related Saudi Arabian outbreak are in boldface. Clades are labeled at their roots and described in the text. Subclade IVa is presented in Fig. 2.

We sequenced the genomes of 92 *N. meningitidis* serogroup W isolates, 9 from CC175 and 83 from CC11 (Table 1; see also Table S1 in the supplemental material). The CC175 genomes had minimal diversity among them and did not show a clear phylogenetic structure (see Materials and Methods; see also Fig. S1). Within CC11, we identified four major subclades (I through IV) based on their association with large epidemics (Fig. 1; Table 2). Subclades I and III did not contain isolates associated with meningitis epidemics. Subclade I consisted of 7 genomes, with the only 2 meningitis belt isolates being from the 1990s. Subclade III consisted of 4 isolates from the meningitis belt from 2001 to 2004. One strain (M07161) isolated in 1994 from Mali did not fit into a major subclade but was basal to subclades III and IV with high bootstrap support (99%).

10.1128/mSphere.00201-16.1 Table S1  Isolate and sequencing details. Subclades for clonal complex 11 are defined in the text and figures. The number of unique hqSNPs was calculated for the Lyve-set alignment. Antigen subtyping information is based on variable regions. Gene allele identifiers were retrieved from PubMLST. Download Table S1, XLSX file, 0.02 MB. Copyright © 2016 Retchless et al. 2016 Retchless et al. This content is distributed under the terms of the Creative Commons Attribution 4.0 International license.

10.1128/mSphere.00201-16.7 Figure S1  Recombination-adjusted phylogeny of 9 single-contig CC175 genomes. ClonalFrameML inferred 95 recombination events, with mean import length (I) of 626 bp, mean divergence (D) of 3.8%, and a relative frequency of recombination to mutation (R) of 0.58. All branches have 100/100 bootstrap support. Download Figure S1, EPS file, 0.03 MB. Copyright © 2016 Retchless et al. 2016 Retchless et al. This content is distributed under the terms of the Creative Commons Attribution 4.0 International license.

**TABLE 1:** Epidemiological context for the isolates analyzed in this study

Country	ST/CC^a^	Yr collected^b^	No. of isolates
Algeria	ST11/CC11	1997	1
		1999	1
		2001	1
Benin	ST2881/CC175	2004	1
		2006	1
		2007	1
Burkina Faso	ST11/CC11	2001	4
		**2002**	**2**
		2004	1
		2011	11
		**2012**	**21**
	ST2961/CC11	2011	1
	ST2881/CC175	2008	1
		2010	1
Cameroon	ST11/CC11	2001	2
Central African Republic	ST11/CC11	2001	2
Djibouti	ST11/CC11	2004	1
Gambia	ST11/CC11	1995	1
Mali	ST11/CC11	1994	2
		2007	3
		2012	21
	ST11407/CC11	2012	1
Mauritius	ST11/CC11	2001	1
Niger	ST11/CC11	2001	1
		2002	1
	ST2881/CC175	2003	1
		2005	1
		2006	1
Saudi Arabia	ST11/CC11	**2000**	**1**
Senegal	ST11/CC11	2002	1
Togo	ST2881/CC175	2007	1
Incomplete data	ST11/CC11	N/A	2
Previously published	ST11/CC11	N/A	4

a ST/CC, sequence type profile/clonal complex identifier, from MLST.

b Boldface indicates years with epidemics and large outbreaks involving CC11.

**TABLE 2:** Observations of isolates from clonal complex 11 subclades^a^

Region and country	No. of isolates from indicated subclade in^c^:															
	1994	1995	1997	1999	2000	2001	2002	2003	2004	2005	2006	2007	2008	2010	2011	2012
Hyperendemic meningitis belt																
Burkina Faso						4 II	**2 II**		1 III				*	*	12 IVa	**22 IVa**
Mali	1 I, 1 NA^b^											1 II, 2 IV				21 IVa
Niger						1 III	1 III	*		*	*					
Nonhyperendemic meningitis belt																
Benin									*		*	*				
Cameroon						2 IV										
Central African Republic						1 III, 1 IV										
Gambia		1 I														
Senegal							1 IV									
Togo												*				
Not meningitis belt																
Algeria			1 I	1 I		1 IV										
Djibouti									1 IV							
Mauritius						1 IV										
Saudi Arabia					**1 IV**											

a Subclades are identified in Fig. 1 and text.

b NA, not applicable; isolate M07161 did not fit into a major subclade.

c Asterisk indicates single observation of CC175 isolate.

Sequence diversity was described further by the number of high-quality single-nucleotide polymorphisms (hqSNPs) between each pair of CC11 isolates in our study (see Table S2 in the supplemental material). After excluding the outgroup, the alignment contained 6,162 variable positions. The diversity within and between each labeled subclade is given in Table 3 (see also Tables S3 and S4).

10.1128/mSphere.00201-16.2 Table S2  Number of hqSNPs distinguishing each pair of genomes. Download Table S2, XLSX file, 0.04 MB. Copyright © 2016 Retchless et al. 2016 Retchless et al. This content is distributed under the terms of the Creative Commons Attribution 4.0 International license.

10.1128/mSphere.00201-16.3 Table S3  Core genome nucleotide diversity within labeled subclades, minimum hqSNP counts between clades, and discriminatory hqSNP counts for each subclade. Download Table S3, XLSX file, 0.01 MB. Copyright © 2016 Retchless et al. 2016 Retchless et al. This content is distributed under the terms of the Creative Commons Attribution 4.0 International license.

10.1128/mSphere.00201-16.4 Table S4  Locations of SNPs that distinguish major clades from each other, according to the reference genome NM3683. SNPs are clustered if they fall into the same phylogenetic class and less than 500 bp separates them. SNPs are categorized as being either hqSNPs or Mauve SNPs and as being either homoplasic or discriminatory. The SNPs included are discriminatory for subclades II, IV, IVa, III/IV, or II/III/IV. Gene annotations from NM3683 are listed if an SNP is contained in the gene. Annotations are based on PubMLST, when available, and otherwise on the Fam18 reference. Download Table S4, XLSX file, 0.03 MB. Copyright © 2016 Retchless et al. 2016 Retchless et al. This content is distributed under the terms of the Creative Commons Attribution 4.0 International license.

**TABLE 3:** Core genome similarity between isolates in the major subclades

Subclade	Range of hqSNP counts or % similarity between indicated subclades^a^				
	I	II	III	IV	IVa
I	26–1,410	**99.97**	**99.96**	**99.97**	**99.95**
II	642–1,488	2–238	**99.98**	**99.98**	**99.97**
III	707–1,467	393–697	20–310	**99.99**	**99.98**
IV	662–1,904	348–1137	132–897	0–1,221	**100**
IVa	938–1,904	637–1,137	417–897	0–1,221	0–684

a Minimum and maximum counts of hqSNPs distinguishing isolates in the subclades are presented on the diagonal and below. Maximum sequence similarity between isolates, based on an alignment of 1,982,813 nucleotides, is presented above the diagonal in boldface. Isolate counts for each subclade are I (*n* = 7), II (*n* = 7), III (*n* = 4), IV (*n* = 67), and IVa (*n* = 55).

### Subclade associated with the epidemic in Burkina Faso during 2002.

Subclade II consists of 2 isolates from the first large *N. meningitidis* serogroup W epidemic in Burkina Faso (2002), 4 isolates from the previous year, and a later isolate from Mali (2007). The epidemic-associated isolates had 21 hqSNPs separating them, greater than the separation between those two and many isolates from 2001, which is as low as 5 hqSNPs (see Table S5 in the supplemental material). This was in agreement with the phylogenetic analysis that depicted these 6 isolates as a closely related group (Fig. 1) that was clearly distinguishable from the isolate recovered in 2004 (subclade III) and the later isolate from Mali in 2007 (subclade II).

Subclade II was distinguished from the others by 128 hqSNPs found in 16 diverse locations in the genome (see Table S4 in the supplemental material). A large cluster of hqSNPs was present in proximity to the *nadC*, *nicA*, and *nicB* genes (PubMLST identifiers [IDs] NEIS1770 to NEIS1773; 96 hqSNPs).

10.1128/mSphere.00201-16.5 Table S5  Core genome similarity of isolates in subclade II, associated with the 2002 Burkina Faso epidemic. Download Table S5, DOCX file, 0.01 MB. Copyright © 2016 Retchless et al. 2016 Retchless et al. This content is distributed under the terms of the Creative Commons Attribution 4.0 International license.

### Subclade associated with the outbreak in Saudi Arabia during 2000.

Subclade IV (*n* = 67) was defined by two isolates from the Hajj-related Saudi Arabian outbreak in 2000 (M07149 and M7124), plus 8 isolates collected from varied locations in Africa between 2001 and 2004. A group of 6 genomes had a maximum of 18 hqSNP differences from each other, each substitution being unique to a single genome (M22722, M22740, M22747, M7124, M07149, and M12752). M07149 had no unique substitutions, indicating that it was very similar to the most recent common ancestor of subclade IV (see Tables S1 and S2 in the supplemental material).

Subclade IV had 24 hqSNPs that distinguished it from other clades (see Table S3 in the supplemental material). These were distributed in 19 genome locations, with small clusters in proximity to the genes *greA* (NEIS1365; 2 hqSNPs) and *lgtA* (NEIS1902; 4 hqSNPs). Inclusion of the SNPs from the Mauve alignment also identified *pglB* (NEIS0399). The clade including subclades III and IV had 91 discriminatory hqSNPs, including one cluster near the *argH* and *galU* genes (NEIS0580 and NEIS0581; 46 hqSNPs), and another near the *nor* and *aniA* genes (NEIS1548; *aniA* is not in PubMLST; 26 hqSNPs). There were no polymorphisms shared among the epidemic subclades (II and IV) that distinguished them from the remainder of the collection.

### Subclade associated with the epidemic in Burkina Faso during 2012.

**FIG 2:**
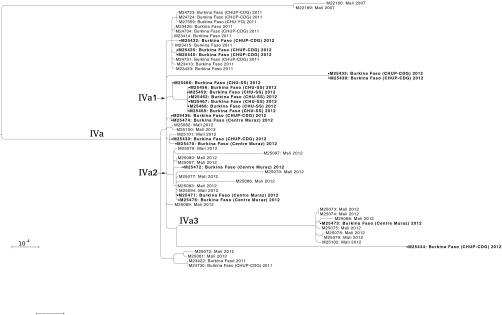
Maximum-likelihood phylogeny of 55 isolates in subclade IVa, rooted on subclade IV. Branches with less than 70% bootstrap support are collapsed. Scale bar represents one substitution per 10,000 bases in the whole-genome alignment. Isolates are labeled with country and year of isolation, with Burkina Faso National Reference Laboratories that provided isolates listed in parentheses. Isolates from Burkina Faso 2012 are in boldface. Subclades mentioned in the text are labeled at their roots.

A nested clade within subclade IV, defined as subclade IVa, comprised 55 isolates from Mali and Burkina Faso in 2011 and 2012 (Fig. 2). Burkina Faso experienced a widespread epidemic in 2012, and all 21 isolates from that year were in subclade IVa. These isolates were subdivided according to the National Reference Laboratories that provided the isolates (see Materials and Methods). Phylogeographic structure within Burkina Faso was evident in some of the clades, but there was also evidence of repeated transmission between countries. For instance, the 7 isolates from Centre Hospitalier Universitaire Sanou Souro (CHU-SS) form a monophyletic group (Fig. 2, subclade IVa1). Of the 22 isolates from Mali, 9 were in subclade IVa2 and 7 in subclade IVa3. The only other isolates in those subclades were from Centre Muraz, which covers districts bordering Mali; of the 6 isolates from Centre Muraz, 3 were in IVa2 and 1 in IVa3. In contrast, the isolates from Centre Hospitalier Universitaire Pédiatrique-Charles de Gaulle (CHUP-CDG) in 2012 were widely dispersed (including the most divergent isolates, M25434 and M25433), consistent with this laboratory receiving isolates from varied locations in Burkina Faso.

Isolates from the 2012 Burkina Faso epidemic had a maximum of 684 hqSNPs between any two isolates (see Table S6 in the supplemental material), accounting for the broad diversity of subclade IVa. Some of these isolates were very similar to isolates collected in Burkina Faso during 2011 (6 hqSNPs) and in Mali during 2012 (1 hqSNP), consistent with the phylogeny, where none of these groups were monophyletic. Subclade IVa was distinguished from other subclades by 240 hqSNPs found in 17 locations on the genome (see Table S4).

10.1128/mSphere.00201-16.6 Table S6  Core genome SNP diversity of isolates in subclade IVa, associated with the 2012 Burkina Faso epidemic. Download Table S6, DOCX file, 0.01 MB. Copyright © 2016 Retchless et al. 2016 Retchless et al. This content is distributed under the terms of the Creative Commons Attribution 4.0 International license.

### Diversity at possible subtyping loci.

**FIG 3:**
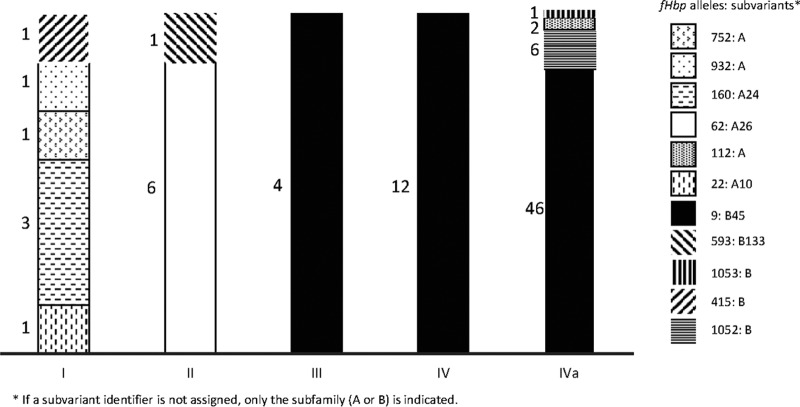
Frequencies of *fHbp* alleles in each subclade of CC11. Each bar represents a different subclade, colored by the proportion of isolates from that subclade that carry each allele, with the number of isolates written to the left. The bar for subclade IV does not include isolates from subclade IVa. Each allele encodes a different protein, and protein subvariants are listed in the legend.

We examined the diversity in several genes that are regularly used to genotype meningococcus (see Table S1 in the supplemental material) (22). The *porA* subtype was uniform within clonal complexes (P1.5,2 in CC11 and P1.5-1,2-36 in CC175), while the *fetA* variable region was largely uniform, with a few low-frequency variants. Sequence variation at the *fHbp* locus has been proposed as a potential marker for the Hajj-related outbreak strain (20); we observed 11 *fHbp* alleles among the CC11 isolates, encoding six subfamily A proteins and five subfamily B proteins (alternatively known as variant groups 2/3 or group 1, respectively). Alleles associated with both subfamilies were found among isolates of subclades I, II, and IVa (Fig. 3).

To identify additional loci that may distinguish the CC11 subclades from each other, we evaluated the PubMLST allele assignments at several loci where hqSNPs distinguish the subclades. A subset of loci for which alleles were strongly associated with subclades is presented in Table 4 (the full set is in Table S4 in the supplemental material); it includes two loci from the ribosomal multilocus sequence type (MLST) scheme. Reflecting this variation, the subclade IV isolates have ribosomal sequence type (rST) 2332 and subclade IVa isolates have rST 7546 (23).

**TABLE 4:** Select loci with SNPs distinguishing subclades, described by their PubMLST allele assignment

Locus^a^	PubMLST ID^b^	Pattern^c^	Allele in subclade^d^					No. of SNPs^f^
			I	II	III	IV^e^	IVa	
*mafS7*	NEIS2090	II	1	18	1	1^g^	1	56
*nadC*	NEIS1770	II	1	237	1	1	1	30
*nor*	NEIS1548	III/IV^h^	149	149	1	1	1	59
*galU*	NEIS0581	III/IV^h^	230	230	351	351	351^i^	36
*nhbA*	NEIS2109	III/IV^h^	17	17	72	72	72	1
*opcB*	NEIS1877	II/III/IV	1	2	2	2^j^	2	2
*folP*	NEIS1609	II/III/IV	14^k^	1	1	1	1	67
*lgtA*	NEIS1902	IV	17^l^	17	17	37^l^	37	2
*lgtB*	NEIS1901	IV	79	79	79	77^m^	77	4
*rpmA*	NEIS1848	IV	1	1	1	4	4	1
*rpsO*	NEIS0552	IVa	1	1	1	1	16	1

a Locus names are taken from PubMLST annotation if available; otherwise, the mapping of Fam18 annotations to the reference sequence is used.

b ID, identifier.

c SNP patterns are categorized by which subclades are distinguished from the outgroup.

d The predominant allele is reported for each subclade; minor alleles are noted in the footnotes.

e Counts for subclade IV do not include isolates from subclade IVa.

f The SNP count is the number of polymorphisms separating the two listed PubMLST alleles.

g *mafS7* is not present in M22765.

h M07161 shares alleles with subclades I/II for *nor* and *galU* but with III/IV for *nhbA.*

i M25432 has *galU* allele 2.

j M22160 and M22189 have *opcB* allele 47.

k M25419 and M10208 have *folP* allele 4.

l *lgtA* is not found in M22772 and Nm3686.

m M22772 has *lgtB* allele 67.

## DISCUSSION

Concern about the epidemic potential of CC11 *N. meningitidis* serogroup W increased greatly following the multinational outbreaks among Hajj pilgrims returning from Saudi Arabia in 2000 (5, 6, 9, 12, 19). The isolates collected from meningitis belt countries in 2001 and 2002 include representatives of three different CC11 *N. meningitidis* serogroup W subclades, one of which (subclade IV) may be descended from the strain that caused the Saudi Arabian outbreak (Table 2). Isolates obtained from the Burkina Faso 2002 epidemic are a separate lineage (subclade II), as are some of the isolates from disease cases outside large epidemics (subclade III). Four isolates from subclades II and III were also sequenced for the study of Lucidarme et al. (8) (M22797/30098, M22759/30087, M22769/30089, and M22745/30104), where they were placed into two clusters labeled as the “Burkina Faso/North African strains.”

While subclade IV was not the only cause of CC11 *N. meningitidis* serogroup W disease in the meningitis belt after 2000, isolates very similar to the Hajj-related outbreak strain were collected in the region. These strains may have been dispersed during the 2000 outbreak, as indicated by the minimal diversity among the subclade IV isolates collected in 7 countries from 2000 to 2004, the absence of phylogenetic structure at the base of subclade IV where 9 branches join (Fig. 1), and the absence of any hqSNPs between the consensus genome sequences of those isolates and the genome of isolate M07149, which was collected during the Saudi Arabian outbreak. Two of these isolates were identified as part of the “Anglo-French Hajj strain” by Lucidarme et al. (8) (M22722/2001076 and M22765/2002029).

The isolates of subclade III were very closely related to the Hajj-related outbreak strain (99.99% sequence identity; minimum of 132 hqSNPs) and share the antigen gene profile of the Hajj-related outbreak strain that was identified by Mustapha et al., specifically the presence of *fHbp* allele 9 (Fig. 3) (20). However, they are distinguished from the Hajj-related outbreak strain at 19 loci where all subclade IV isolates have derived variants (see Table S4 in the supplemental material), indicating that subclade III isolates are not derived from the Hajj-related outbreak strain. They are further distinguished from the subclade IV strains by their greater sequence diversity and the presence of phylogenetic structure within the clade (Fig. 1), which is evident even when recombination is accounted for (see Fig. S2). Altogether, this indicates that subclade III did not undergo the same population dynamics as the strains that make up subclade IV and is a separate linage.

10.1128/mSphere.00201-16.8 Figure S2  Recombination-adjusted phylogeny of 41 single-contig CC11 genomes, rooted on NM3683. ClonalFrameML inferred 480 recombination events (279 when the outgroup branch is excluded), with mean import length (I) of 641 bp, mean divergence (D) of 4.4%, and a relative frequency of recombination to mutation (R) of 0.60. Branches with less than 75/100 bootstrap support are collapsed. Download Figure S2, EPS file, 0.1 MB. Copyright © 2016 Retchless et al. 2016 Retchless et al. This content is distributed under the terms of the Creative Commons Attribution 4.0 International license.

Isolates from the Burkina Faso epidemic of 2002 comprised a distinct subclade (II), which clearly diverged from subclades III and IV prior to the 2000 Saudi Arabian outbreak. This was demonstrated by the presence of an isolate from 1994 (M07161) being placed on the lineage leading to subclades III and IV with high confidence (bootstrap value of 99%). In the years after the 2002 epidemic, CC11 *N. meningitidis* serogroup W was rarely identified among either disease isolates or carriage isolates (11, 24, 25) until isolates from subclade IVa were recovered in 2011 and 2012. This is consistent with the “clonal wave” model of meningococcal strain replacement in meningitis belt countries and communities (26). 

Isolates recovered during the 2012 epidemic in Burkina Faso belonged to subclade IVa, a lineage resulting from the clonal expansion and international dispersion of subclade IV that coincided with the Hajj-related Saudi Arabian outbreak in 2000. This subclade contains a different ribosomal MLST profile (7546) than any isolates examined by Lucidarme et al. (8). The geographic location of the subclade IVa ancestral lineage between 2000 and 2011 cannot be inferred from the isolate collection in this analysis, which primarily includes western meningitis belt countries in which a previous study identified low frequencies of CC11 *N. meningitidis* serogroup W isolates from 2005 to 2010 (11). One possibility is that this lineage was only introduced to the western meningitis belt shortly before 2011; alternatively, a local population may not be represented in this analysis. The phylogeographic structure within subclade IVa indicated that most transmission is geographically restricted during epidemics, but repeated pathogen transmission has still occurred across the border of Mali and Burkina Faso, as indicated by the phylogenetic mixing of isolates from Mali in 2012 with those collected in Burkina Faso in 2011 and 2012 (Fig. 2). Additional isolates from this clade were collected in Niger during 2015 (27), demonstrating that CC11 *N. meningitidis* serogroup W populations are established in the meningitis belt.

### Conclusion.

The *N. meningitidis* serogroup W isolates associated with the epidemics in Burkina Faso during 2002 and 2012 belong to two distinct subclades, closely related to CC11 isolates recovered from sporadic invasive meningococcal disease cases in meningitis belt countries since 2000. The subclade associated with the Burkina Faso epidemic of 2012 was descended from the Hajj-related outbreak strain, which became globally dispersed near the time of the Hajj-related Saudi Arabian outbreak in 2000. This subclade included all *N. meningitidis* serogroup W isolates examined that were collected from Mali and Burkina Faso in 2011 and 2012, but it included none of the isolates from the previous decade. These results, along with a large epidemic in Niger during 2015 caused primarily by a *N. meningitidis* serogroup C strain first identified in 2013 (27), indicate that meningococci can spread rapidly to cause large epidemics, stressing the importance of maintaining *N. meningitidis* surveillance throughout meningitis belt countries following the MACV implementation. The application of whole-genome sequencing to a greater proportion of representative disease and carriage isolates will allow high-resolution tracking of pathogen dissemination at the scale of both countries and continents, detecting epidemic-associated strains as they become established in new districts and generating hypotheses regarding paths of transmission.

## MATERIALS AND METHODS

### Strain selection.

A total of 92 isolates from the Centers for Disease Control and Prevention (CDC) culture collection were sequenced for this analysis; 85 originated from the meningitis surveillance systems of 10 meningitis belt countries (Table 1). Another 7 CC11 isolates from other regions were sequenced, and 4 previously published genomes included, to provide a global context for the diversity of meningitis belt populations (Table 1; see also Table S1 in the supplemental material). The WHO Collaborating Centre in Marseille contributed 20 of these isolates. Isolates were selected first to maximize the temporal and geographic diversity of the data set and second to focus on three notable epidemics: Saudi Arabia 2000, Burkina Faso 2002, and Burkina Faso 2012. Isolates from Burkina Faso were identified with the name of the National Reference Laboratory that provided the isolate, when available. These were Centre Hospitalier Universitaire Pédiatrique-Charles de Gaulle (CHUP-CDG), Centre Hospitalier Universitaire Yalgado de Ouagadougou (CHU-YO), Centre Hospitalier Universitaire Sanou Souro (CHU-SS), and Centre Muraz. The serogroup phenotype was confirmed using slide agglutination (28).

### Genome sequencing.

Pacific Biosciences (PacBio) RSII sequencing was completed for 48 isolates, using P4-C2 sequencing chemistry. Sequences were assembled using PacBio’s Hierarchical Genome Assembly Process version 3 (29), where 30 Mb of the longest corrected reads was used for the initial assembly (see Table S1 in the supplemental material for details). Contiguous sequence (“contig”) circularity was evaluated by identifying repeats at the ends of the single contig, removing the repeat from one end, transferring the sequence from the 3′ to the 5′ end, and assessing whether the manual join point was supported by remapped reads.

An additional 44 isolates were sequenced on an Illumina HiSeq2500 (or MiSeq) instrument to examine the bacterial diversity during epidemics. Illumina sequencing libraries were prepared from extracted DNA by first shearing it to 600 bp using a Covaris LE220 focused ultrasonicator (Covaris, Inc., Woburn, MA). The sheared DNA was processed with the NEBNext ultra DNA library preparation kit following the manufacturer’s protocol (New England Biolabs, Ipswich, MA), using dual barcoding indices. These libraries were paired end sequenced, using TruSeq rapid SBS (sequencing by synthesis) chemistry, with either 100 bp or 250 bp at each end (see Table S1 in the supplemental material). Base calling and demultiplexing were completed with Casava (version 1.8.2). Reads were filtered to have an expected error rate of <1% (Qual = 20) and assembled by SPAdes (version 3.5) (30), discarding small contigs (<300 bp) or those with low coverage (<10×).

### Genome alignment and phylogenetics.

The published genome sequence of NM3683 was used both as a reference for the sequence alignment and as an outgroup to root the phylogeny of CC11. This isolate was collected in Canada in 1970 and previously shown to be an outgroup to the extant CC11 *N. meningitidis* serogroup W population (20). To identify high-quality single-nucleotide polymorphisms (hqSNPs) for phylogenetic analysis, PacBio assemblies and Illumina read sets were simultaneously aligned to the reference genome using Lyve-Set 1.0 (31). SNPs less than 3 bp apart were excluded, as were any base positions with ambiguous characters. The final alignment covered 90.2% of the reference genome and contained 7,384 variable positions and 1,975,429 invariant positions (see Table S2 in the supplemental material). RAxML 8.1.17 (32) was used to generate a phylogeny, using the GTRGAMMAX model with the Stamatakis ascertainment bias correction and 100 bootstraps. Extended majority rule trees constructed from replicate bootstrap sets differed by <5% weighted Robinson-Foulds distance (33). To identify regions with many mutations, all 41 complete genomes were aligned with progressiveMauve (34), using a hidden Markov model (HMM) identity of 95%, identifying 16,419 SNPs in the core genome alignment (i.e., where all genomes contained a base). A progressiveMauve (34) alignment of the 9 CC175 single-contig genomes identified 3,026 SNPs in the core genome alignment. ClonalFrameML (35) was used to account for recombination in a phylogeny constructed from these alignments of single-contig genomes (see Fig. S1 and S2 in the supplemental material). Custom scripts for evaluation of sequence data and phylogenies used BioPython (36).

MLST alleles were identified by BLAST searches of PubMLST allele lists against the assembled genomes (37). Other genes were identified based on the PubMLST annotation of NM3683 where available and, where it was not available, based on the Fam18 NeMeSys (38) annotation, which was transferred to NM3683 by using Rapid Annotation Transfer Tool (RATT) (39). Discriminatory SNPs are those for which variants correspond to monophyletic groups of isolates, distinguishing the specified clade. Homoplasic SNPs are those for which variants are found in polyphyletic groups of isolates.

### Accession number(s).

The genomic data are available in the GenBank database under BioProject accession number PRJNA319252, and individual accession numbers are listed in Table S1 in the supplemental material.
