# Supplementary material for: The Establishment and Diversification of Epidemic-Associated Serogroup W Meningococcus in the African Meningitis Belt, 1994 to 2012
Source: mSphere. 2016 Nov 16;1(6):e00201-16. doi: 10.1128/mSphere.00201-16 (PMC5112335; doi:10.1128/mSphere.00201-16)
Supplement: Table S5 [file sph006162189st5.docx]

Supplemental Table 5: Sequence similarity of isolates in subclade II, associated with the 2002 Burkina Faso epidemic.

|  | Burkina Faso 2001 | Burkina Faso 2002 | Mali 2007^a^ |
| --- | --- | --- | --- |
| Burkina Faso 2001 | 2-19 | **100.00%** | **99.99%** |
| Burkina Faso 2002 | 5-30 | 21 | **99.99%** |
| Mali 2007 | 226-238 | 216-229 | N/A |

Minimum and maximum counts of hqSNPs distinguishing isolates in the groups are presented on the diagonal and below. Maximum sequence similarity between isolates, based on an alignment of 1,982,813 nucleotides is presented above the diagonal. Isolate counts for each sampling period are: Burkina Faso 2001 (n=4), Burkina Faso 2002 (n=2), Mali 2007 (n=1).

1. Two isolates from Mali 2007 are in subclade IV and not evaluated here. Comparisons among a single isolate are not applicable (N/A)
