# Supplementary material for: The Establishment and Diversification of Epidemic-Associated Serogroup W Meningococcus in the African Meningitis Belt, 1994 to 2012
Source: mSphere. 2016 Nov 16;1(6):e00201-16. doi: 10.1128/mSphere.00201-16 (PMC5112335; doi:10.1128/mSphere.00201-16)
Supplement: Table S6 [file sph006162189st6.docx]

Supplemental Table 6: SNP diversity of isolates in subclade IVa, associated with the 2012 Burkina Faso epidemic.

|  | Burkina Faso 2011 | Burkina Faso 2012 | Burkina Faso 2012 | Burkina Faso 2012 | Burkina Faso 2012 | Mali 2012 |
| --- | --- | --- | --- | --- | --- | --- |
|  | Entire country | Entire country | CHU-SS | CHUP-CDG | Centre Muraz | Entire country |
| Burkina Faso 2011 |  |  |  |  |  |  |
| Entire country | 1-67 |  |  |  |  |  |
| Burkina Faso 2012 |  |  |  |  |  |  |
| Entire country | 6-456 | 0-684 |  |  |  |  |
| CHU-SS | 21-91 | 0-437 | 0-26 |  |  |  |
| CHUP-CDG | 6-456 | 0-684 | 8-437 | 0-684 |  |  |
| Centre Muraz | 19-315 | 2-633 | 8-298 | 2-633 | 3-287 |  |
| Mali 2012 |  |  |  |  |  |  |
| Entire country | 21-337 | 1-657 | 10-301 | 8-657 | 1-423 | 0-445 |
|  | | | | |  |  |

Minimum and maximum counts of hqSNPs distinguishing isolates in groups are presented on the diagonal and below. Isolate counts for each sampling period are: Burkina Faso 2011 (n=12), Burkina Faso 2012 (n=21), CHU-SS (n=7), CHUP-CDG (n=8), Centre Muraz (n=6), Mali 20012 (n=22). The maximum sequence similarity in each comparison exceeds 99.99%.
